# Senescent endothelial cells promote liver metastasis of uveal melanoma in single-cell resolution

**DOI:** 10.1186/s12967-024-05430-1

**Published:** 2024-07-01

**Authors:** Liang Ma, Xiaoyu He, Yidian Fu, Shengfang Ge, Zhi Yang

**Affiliations:** 1 Department of Ophthalmology, Ninth People’s Hospital, Shanghai Jiao Tong University School of Medicine, Shanghai, 200025 China; 2 Shanghai Key Laboratory of Orbital Diseases and Ocular Oncology, Shanghai, 200025 China

**Keywords:** Uveal melanoma, KLF4, Endothelial dysfunction, Cellular senescence, Single cell RNA-seq

## Abstract

**Background:**

Uveal melanoma (UM), the most common adult intraocular tumor, is characterized by high malignancy and poor prognosis in advanced stages. Angiogenesis is critical for UM development, however, not only the role of vascular endothelial dysfunction in UM remains unknown, but also their analysis at the single-cell level has been lacking. A comprehensive analysis is essential to clarify the role of the endothelium in the development of UM.

**Methods:**

By using single-cell RNA transcriptomics data of 11 cases of primary and liver metastasis UM, we analyzed the endothelial cell status. In addition, we analyzed and validated ECs in the in vitro model and collected clinical specimens. Subsequently, we explored the impact of endothelial dysfunction on UM cell migration and explored the mechanisms responsible for the endothelial cell abnormalities and the reasons for their peripheral effects.

**Results:**

UM metastasis has a significantly higher percentage of vascular endothelial cells compared to in situ tumors, and endothelial cells in metastasis show significant senescence. Senescent endothelial cells in metastatic tumors showed significant Krüppel-like factor 4 (KLF4) upregulation, overexpression of KLF4 in normal endothelial cells induced senescence, and knockdown of KLF4 in senescent endothelium inhibited senescence, suggesting that KLF4 is a driver gene for endothelial senescence. KLF4-induced endothelial senescence drove tumor cell migration through a senescence-associated secretory phenotype (SASP), of which the most important component of the effector was CXCL12 (C-X-C motif chemokine ligand 12), and participated in the composition of the immunosuppressive microenvironment.

**Conclusion:**

This study provides an undesirable insight of senescent endothelial cells in promoting UM metastasis.

**Supplementary Information:**

The online version contains supplementary material available at 10.1186/s12967-024-05430-1.

## Graphic Abstract

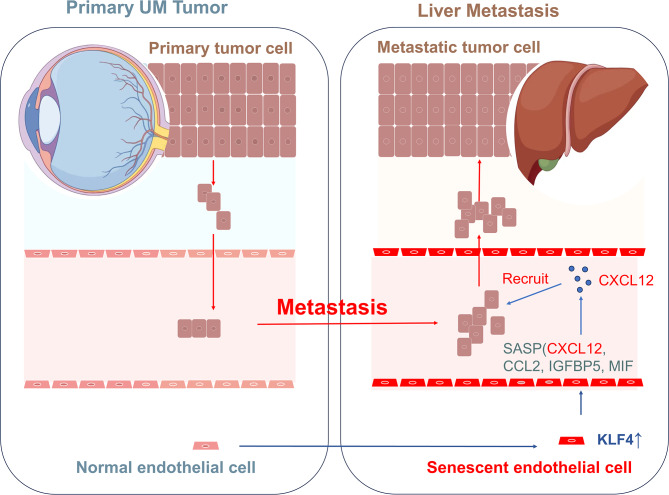

## Background

Endothelial cells (ECs) exert a crucial role within the tumor environment, significantly impacting tumor development and progression. ECs are arranged in a single layer on the inner surface of blood vessels. In addition to controlling the exchange of gases and metabolites between blood vessels and tissues, ECs also regulate hemodynamics, coagulation, angiogenesis, and inflammation [1]. Tumor growth heavily relies on the development of new blood vessels to meet its nutrient demands, and hematogenous metastasis depends on the interaction with endothelial cells. Consequently, the condition of the vascular endothelium holds significant importance, making endothelial cell damage a subject of concern. Such damage can result from factors such as oxidative stress, inflammation, hyper-glycemia, and aging [2]. In our study, we found that endothelial cells exhibit significant senescence during the metastasis of uveal melanoma, which may be associated with liver metastasis of uveal melanoma.

Cellular senescence has an impact on tumor development and progress. The most important features of senescent cells include cell cycle arrest, increased cell size, chromosomal abnormalities, and significant up-regulation of senescence-associated markers, among other characteristics [3]. However, the connection between ECs and tumor cells remains undisclosed. Senescent cells have a significant impact on their surrounding environment. Senescent cells have a significant impact on the surrounding environment [4], because they secrete large amounts of SASP, which includes inflammatory factors, chemokines and matrix proteins that attract peripheral immune cells and cause chronic inflammation, which often leads to an increase in the invasiveness of tumor cells, and more recently, it has been found that senescent endothelial cells induce metastasis of tumor cells [5]. We have confirmed the presence of endothelial senescence in metastatic tumors at the single-cell level.

UM is known for its high malignancy, propensity for metastasis, and lack of effective therapeutic drugs [6]. Traditionally, uveal melanoma is considered a non-immunogenic tumor. However, evidence has shown that uveal melanoma is not devoid of immune cell infiltration. The main reason for the poor immune efficacy is attributed to the specificity of the immune tolerance mechanism [7]. By analyzing single-cell RNA sequencing databases, we discovered that senescent endothelial cells can attract immune cells, especially Treg cells, which further revealed the tumor microenvironment of uveal melanoma.

In this study, we emphasize the crucial interaction between endothelial cells, senescence, and the immune response in the context of uveal melanoma, ultimately paving the way for new research avenues and potential therapeutic strategies.

## Materials and methods

### Cell culture

The cell lines 92 − 1 were from human UM supplied by Professor John F. Marshall (Centre for Tumor Biology, Queen Mary University of London, London, UK). The cell lines OMM2.3 were provided by Professor Martine J. Jager (Department of Ophthalmology, Leiden University Medical Center, Leiden, the Netherlands). 92 − 1 and OMM2.3 cells were cultured in Gibco RPMI 1640. All cells were cultured in media with streptomycin (100 mg/mL), penicillin (100 U/mL), and 10% fetal bovine serum (Gibco) with 5% CO2 at 37 °C.

### Cell transfection

Purchase small interfering RNA (siRNA) and corresponding interfering siRNA specific to KLF4 from GE Biotechnology (Guangzhou, China), and then transiently transfect them into HUVECs using lipo2000 (Invitrogen, Shanghai, China). In short, HUVECs are inoculated into 96 well or 24 well plates 6 h before transfection. When the cell reach-es 50–60% confluence, SiRNA transfection is performed. 48 h after transfection, the transfection efficiency was monitored by RT-PCR or Western blot. HUVEC specific siRNA targets the following sequences in HUVEC mRNA: 5 ‘- GCA GCU UCA CCU AUC CGA U -3’.

### Plasmid construction and transfection

The human KLF4 gene was cloned into the pCDNA3.1(+) expression vector (Invi-trogen). The plasmid construct was verified by DNA sequencing to ensure the correct insertion and sequence of the KLF4 gene. HUVECs were seeded in 6-well plates at a density of 2 × 10^5 cells per well and allowed to reach 70–80% confluence. Transfection was performed using Lipofectamine 3000 (Invitrogen) according to the manufacturer’s instructions. Briefly, 2 ug of the KLF4 expression plasmid or the empty vector (as a con-trol) was diluted in 125 ul of Opti-MEM I Reduced Serum Medium (Gibco). Separately, 5 ul of Lipofectamine 3000 was diluted in 125 ul of Opti-MEM. The diluted DNA and Lipofectamine solutions were combined and incubated for 5 min at room temperature. The DNA-Lipofectamine complexes were then added dropwise to the cells.

### SA-β-gal staining

At indicated times, cultured HUVECs were washed with PBS and fixed in staining fixatives for 15 min at room temperature. Fixed cells were stained with fresh SA-β-gal staining solution 37 °C overnight according to manufacturer’s protocol (G1580, Solarbio Life Science).

### Transwell

A 24-well Transwell system with 8‐µm pore size polycarbonate filters (Corning) was used to detect the migration ability of uveal melanoma and conjunctival melanoma cells. To the upper chamber, 1.0 × 104‐1.0 × 105 tumor cells suspended in 200‐250 µL culture medium with 2% FBS were added, while the lower chambers were filled with 600‐650 µL culture medium with 10% FBS. After incubation at 37 °C for 24 h, cells were fixed with methanol and stained with 1% crystal violet (C8470, Amresco, San Diego, CA, USA). The cells in the upper chambers were removed, and those that migrated to the lower chambers were photographed by ECLIPSE Ti inverted microscope system (Nikon Precision, Shanghai, China) and counted by ImageJ software.

### Western blot

Western blotting was performed as previously described [8]. Briefly, the polyvinylidene fluoride membranes (Merck-Millipore, Shanghai, China) with the transferred proteins were first incubated with a primary antibody overnight at 4 °C, and then with a secondary antibody conjugated to a fluorescent tag or a horseradish peroxidase (HRP) tag. The band signals were tested using an Odyssey infrared imaging system (LI‐COR, Lincoln, NE, USA) or a chemiluminescence imaging system (Tanon, Shanghai, China). Antibodies against the following antigens were used: cyclin dependent kinase inhibitor P21 (1:1,000, 10355-1-AP, proteintech), Krüppel-like factor 4 (KLF4, 1:2,000, ab215036, Abcam), and β-actin (1:2,000, #4967, CST).

### RNA extraction, reverse transcription, and qPCR

Total RNA of ocular melanoma cells and normal cells was extracted using an EZpress RNA purification kit (B0004, EZBioscience, Beijing, China). Reverse transcription was performed using a PrimeScript RT-PCR kit (Takara Biotechnology, Dalian, Liaoning, China). qPCR was performed using a standard SYBR Green PCR kit (Applied Biosystems, Thermo Fisher Scientific) according to the manufacturer’s instructions. GAPDH was used as an internal control.

### Immunofluorescence

Tissues embedded in paraffin were deparaffinized, rehydrated, fixed, and blocked with 5% normal goat serum, and were then incubated with the primary antibody KLF4 (1:400, ab215036, Abcam) and CD31 (1:400, ab9498, Abcam) at 4 °C overnight. Next, the tissue slides were incubated with secondary antibodies for 60 min, and 4’,6-diamidino‐2‐phenylindole (DAPI, Servicebio, G1012) was used to counterstain nuclei for 5 min. Digital images were acquired using a Pannoramic MIDI (3DHISTECH, Hungary).

### scRNA-seq data processing and analysis

The data that support the findings of this study are openly available in GEO: GSE138433, GSE176029, and GSE139829. The 10× scRNA-seq data were processed as follows: (1) R package “Seurat” was used to convert the 10× scRNA-seq data into Seurat objects [9]; (2) original counts were checked for quality by calculating the proportion of mitochondrial or ribosomal genes and eliminating cells with low quality; (3) after quality control, “FindVariableFeatures” function was used to screen the top 2000 highly variable genes; (4) based on 2000 genes for principal component analysis (PCA), uniform manifold approximation and projection (UMAP) [2] was used for dimensionality re-duction and cluster identification; (5) using the “Find All Markers” function, log2 [Fold-change (FC)] was set to 0.5 and min, and pct was set to 0.25 to identify markers in differ-ent clusters; (6) using the “FindMarkers” function, log2 [Foldchange (FC)] was set to 0.5 and min, and pct was set to 0.25 to identify DEG of endothelial cells came from meta-statics or primary, as well as DEG between different levels of senescence in endothelial cells; (7) the overall difference in gene function among the different clusters was investigated by the “GSVA” package using the “c2. cp.kegg.v7.2. symbols.gmt” gene set (8) The “monocle” package [10] scans cell trajectories and pseudotime distributions and re-duces dimensionality using the “DDRTree” approach. (9) Cell-cell communication analysis and network visualization were finally performed using the “CellChat” [11] and “patchwork” software packages.

### KEGG functional enrichment analysis

For gene set functional enrichment analysis, we use the KEGG rest API(https://www.kegg.jp/kegg/rest/keggapi.html) We obtained the latest KEGG Pathway gene annotation as the background and mapped the genes to the background set. We used the R software package ClusterProfiler (version 3.14.3) for enrichment analysis to obtain the results of gene set enrichment. Set the minimum gene set to 5 and the maxi-mum gene set to 5000, *P* value of < 0.05 and a FDR of < 0.25 were considered statistically significant.

### Immune cells infiltration

We accessed the Uveal Melanoma (UM) dataset from The Cancer Genome Atlas (TCGA) using SANGERBOX (http://www.sangerbox.com/), a comprehensive online platform for bioinformatics analysis. SANGERBOX provides user-friendly access to TCGA datasets, facilitating the retrieval of genomic and clinical data for various cancers, including UM. And downloaded the unified and standardized UM dataset (*N* = 80) from the database, and further divided each sample into two groups based on whether the long-term event is a distant metastasis: Primary and Metastasis. Deconvolve bulk RNA using the LM22 file of CIBERSORTx (https://cibersortx.stanford.edu/).

### Statistical analysis

GraphPad Prism software (version 8.0) was used for the statistical analyses. Data are presented as mean ± standard deviation (SD), mean ± standard error of mean (SEM), or median with interquartile range as required, and an unpaired two-tailed Student’s t‐test was used to assess the differences between the two groups. Survival plots were depicted with Kaplan‐Meier curves, and *P* values were calculated with the log‐rank test. *P* value < 0.05 was considered statistically significant.

## Results

### scRNA-seq reveals a larger population of ECs in metastatic uveal melanoma

**Fig. 1:**
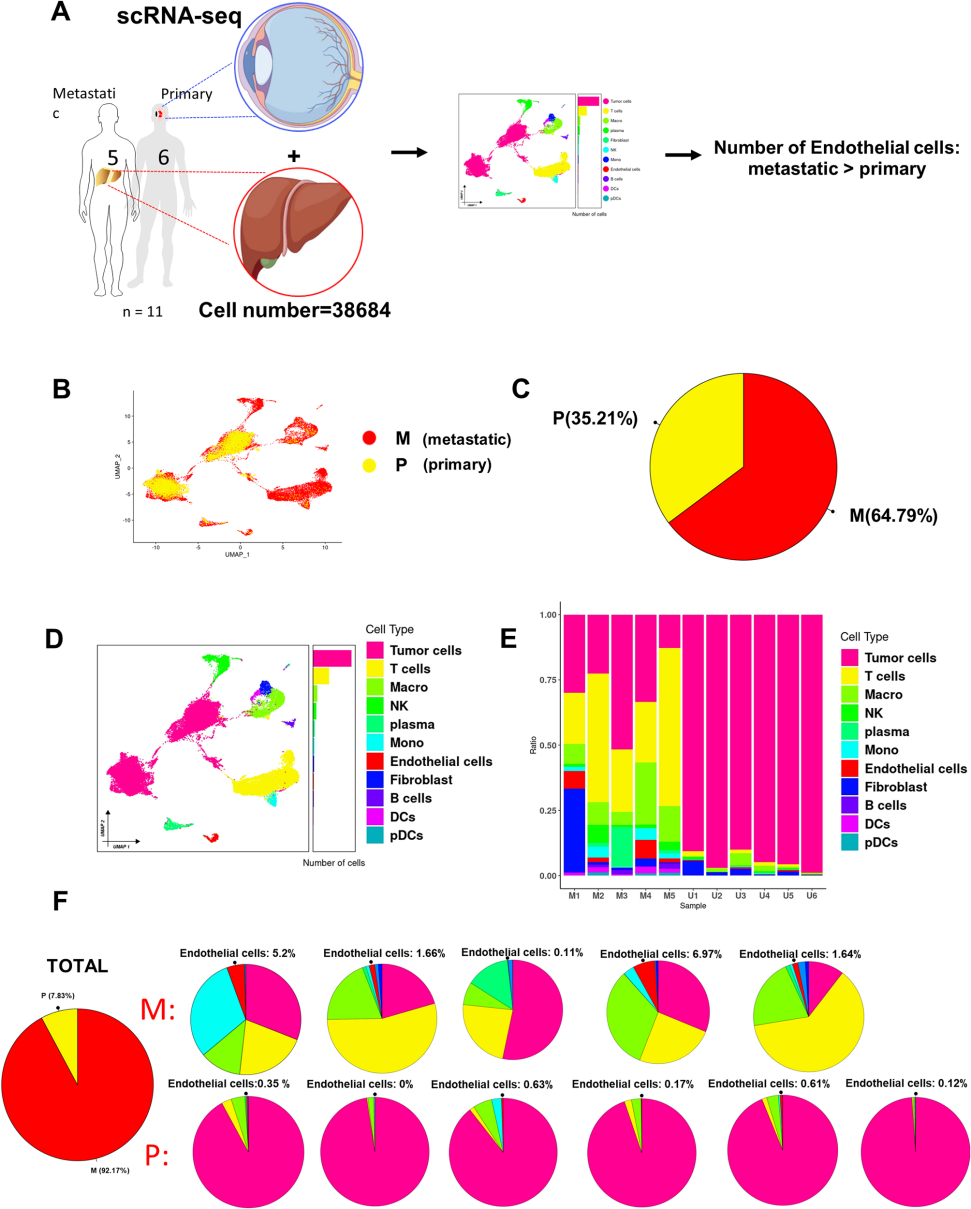
The single cell RNA-seq atlas of endothelial cell in primary and metastatic UM. (**A**) Experimental and data analysis workflow for scRNA-seq and bulk seq. (**B**) UMAP diagram of uveal melanoma differentiated by metastasis or non-metastasis. (**C**) Percentage of metastatic versus non-metastatic uveal melanoma. (**D**) UMAP representation of uveal melanoma classes labeled with cell types. (**E**) Cell proportions in different samples of uveal melanoma. (metastasis samples (M1-M5); primary samples (U1-U6)). (**F**) Percentage of endothelial cells in each sample. (Upper: five metastasis samples (M1-M5); Bottom: six primary samples (U1-U6))

We downloaded the transcriptome data of three 10X single-cell sequencing of GSE138433 [12], GSE176029 [9], and GSE139829 [6] from the GEO database, in which GSE139829 was taken from metastatic samples only, and obtained a sample cohort of six in situ uveal melanomas with five liver metastasis from uveal melanomas. After data merging, filtering, dimensionality reduction, de-batching, and unsupervised cell clustering [2, 10, 11], we obtained 11 cell types and 38,684 cells (Fig. 1A). Tumor cells were identified using MLANA, MITF. The following cell types were annotated using: T Cells (CD3D, CD3E, CD8A), B cells (CD19, CD79A, MS4A1 [CD20]), Plasma cells (IGHG1, MZB1, SDC1, CD79A), DCs (CLEC9A, CD1C, CD1E), pDCs (GZMB), Mono (Monocytes; S100A8, S100A9, S100A12), Macro (Macrophages; CD68, CD163), NK (NK Cells; NKG7, GNLY, KLRD1), Fibro (Fibroblasts, FGF7), and Endothelial cells (PECAM1, VWF). The cells were presented as unified manifold approximation and projection (UMAP) plots to show the specific cellular profiles (Fig. 1D), and we subsequently compared the difference in endothelial cell numbers between in situ and metastatic cancers (Fig. 1B, 1 C), and found that the number of endothelial cells was much lower in carcinoma in situ than in metastatic carcinoma (Fig. 1E), and a subsequent detailed comparison of the proportion of endothelial cells in each of the 11 samples confirmed that the number of endothelial cells was much higher in metastatic tumors than in situ tumors (Fig. 1F).

### ECs within metastatic UM have a significant tendency to senescence

**Fig. 2:**
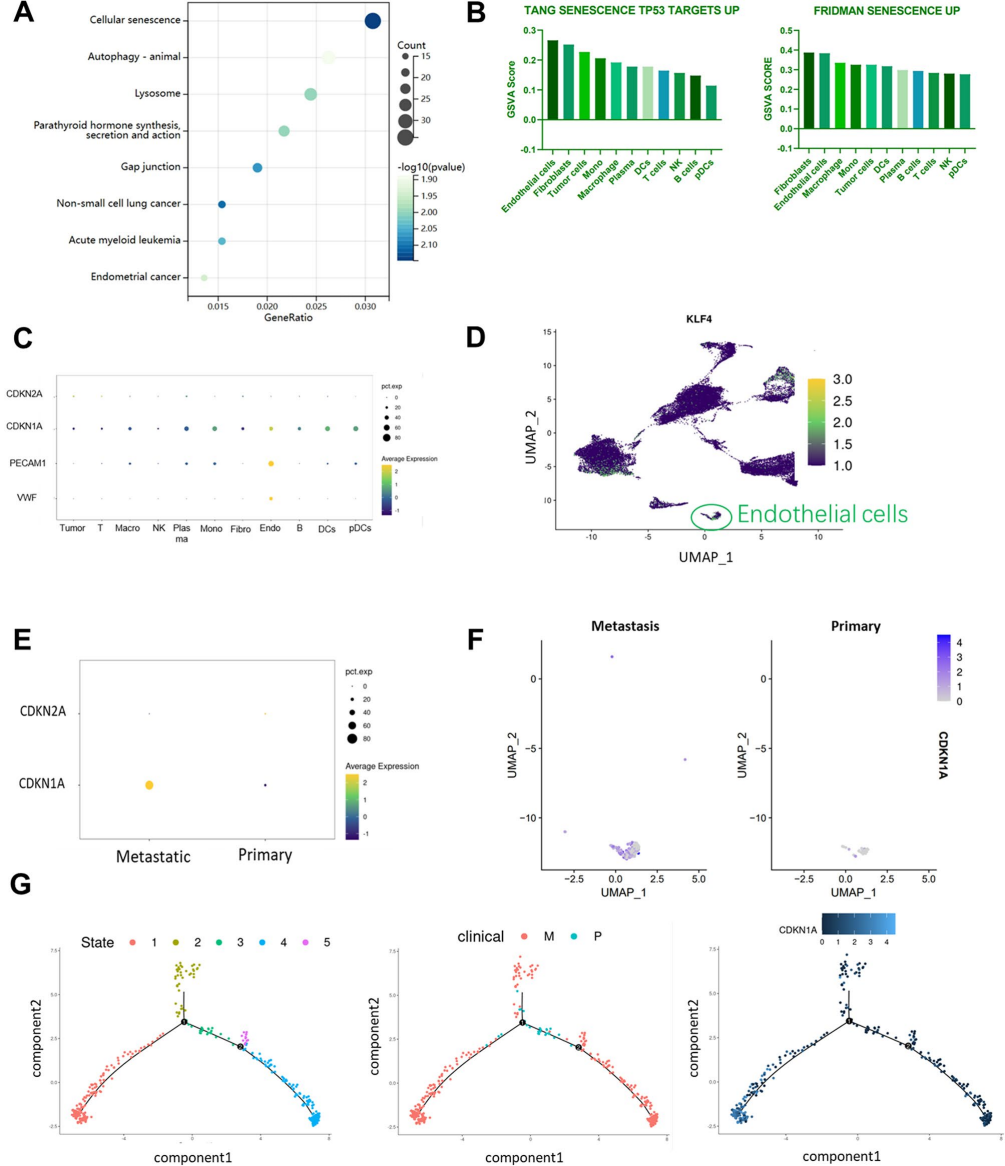
Significant senescence status of endothelium in metastatic UM. (**A**) Dot plots showing the DEGs between metastatsis and primary significant enrichment into cellular senescence. (**B**) Bar graph showing the scoring of the senescence pathway in different cell types (GSVA). (**C**) Dot plot showing significantly higher CDKN1A expression in endothelial cells than in other cell types. (**D**) UMAP demonstrating the distribution of KLF4 in the UM. (**E**) Dot plot demonstrating that CDKN1A was significantly higher in metastatic endothelial cells than in primary endothelial cells. (**F**) UMAP showing that CDKN1A was significantly higher in metastatic endothelial cells than in primary endothelial cells. (**G**) Pseudotime demonstrates specific expression of CDKN1A in metastatic endothelial cells (M: metastasis; P: primary), CDKN1A is higher in state 1 which mainly composed by endothelial cells of metastasis tumor

Endothelial cells are getting more and more attention, and the effect of endothelial cell injury on the biological behavior of tumors is also crucial, and we tried to explore what biological function differences between the endothelial cells of metastatic tumors and in situ tumors [13]. We performed KEGG analysis of DEG (differential expression genes) between metastatic tumor endothelial cells and tumor endothelial cells in situ and found that differential genes could be significantly enriched into the cellular senescence pathway (Fig. 2A). To further investigate the potential role of cellular senescence in uveal melanoma metastasis, suggesting a potential link between cellular senescence and cancer metastasis [8, 14]. To support this thought and explore the overall senescence status of the tumor and tumor microenvironment, we conducted senescence scoring using the FRIDMAN SENESCENCE UP and TANG SENESCENCE TP53 UP [15–17] gene sets (Fig. 2B). We observed that endothelial cells exhibited one of the highest senescence scores among all cell clusters, along with fibroblasts. This suggests that endothelial cells may display the highest level of senescence in the samples [18]. It was found that endothelial cells were one of the most significantly senescent clusters in the sample as a whole, and only fibroblasts had similar senescence scores to endothelial cells, which can be assumed to exhibit the highest level of senescence in the sample. We then compared the expression levels of the senescence genes P16,P21 in the overall sample and found that the difference in expression between P16 was not significant in the overall sample [19], but the expression of P21 was abnormally elevated in endothelial cells (Fig. 2 C, 2D). We divided the endothelial cells into two parts according to the sample source and compared the P21 expression in metastasis and in situ, and the endothelial cells in metastatic tumors had significantly higher P21 expression than in situ tumors [19]. We suggest that there may be significantly more senescent endothelial cells in metastasis than in situ tumors (Fig. 2E, 2 F). According to the current understanding of senescence [3, 20], the role of senescent endothelium in promoting tumor metastasis has been validated in other cancers, and SASP secreted by senescent cells significantly promotes the inflammatory state of tumor tissues while stimulating the proliferation and migration of tumor cells [21], whereas it is highly likely that the significantly increased senescent endothelium in metastatic tumors is involved in the metastasis of uveal melanoma. To confirm the reliability of this analysis, we performed a proposed time-series analysis using monocle2 [22] to group endothelial cells according to samples, and it was seen that the elevated P21 was mainly concentrated in post-metastatic endothelial cells (Fig. 2G).

Combining the high senescence score of endothelial cells in GSVA with the high expression of P21 relative to other cell types, we concluded that many senescent cells exist in the endothelium. Endothelial senescence is a type of endothelial injury, and senescent endothelial cells have significant effects on the tumor microenvironment within the tumor, including increasing tumor stemness, promoting tumor migration, and regulating the immune environment. However, we do not know exactly how endothelial senescence occurs.

### KLF4 is a potential driver factor for endothelial senescence

**Fig. 3:**
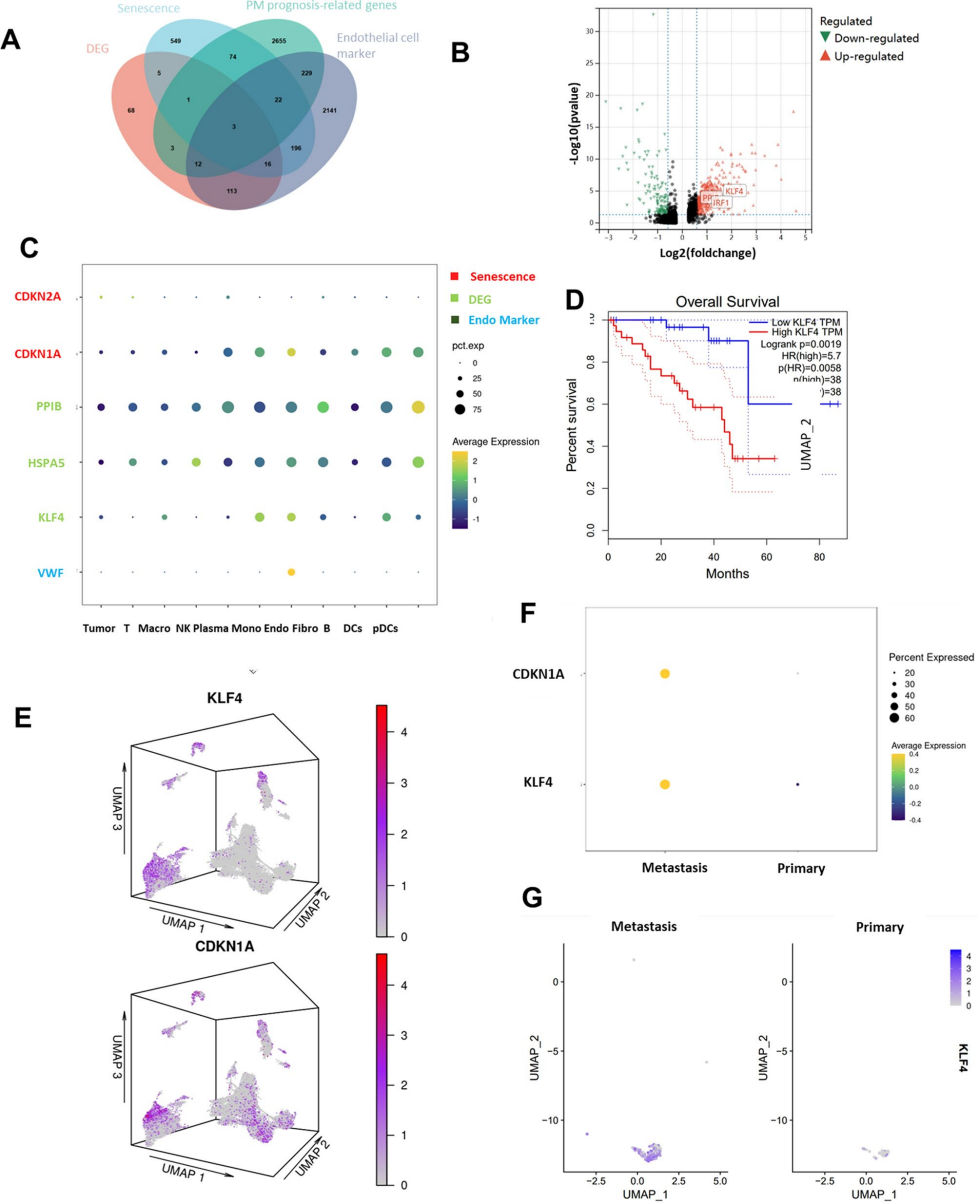
Identification of KLF4 as the potential senescent driver of ECs in metastatic UM. (**A**) Venn diagram the overlap (HSPA5, KLF4, PPIB) between DEG, senescence, UM prognosis-related gene and endothelial cell marker. (**B**) Volcano map showing expression of genes in overlap (HSPA5, KLF4, PPIB) in endothelial cells of DEG. (**C**) Dot plot demonstrated that KLF4 is enriched in endothelial cells. (**D**) Prognosis of KLF4 in UM. (**E**) Three-dimensional distribution maps in UMAP demonstrate the similarity of KLF4 and CDKN1A distribution. (**F**) Dot plot displaying the expression of CDKN1A and KLF4 between metastasis and primary in endothelial cells. (**G**) UMAP showing that KLF4 was significantly higher in metastatic endothelial cells than in primary endothelial cells. Color scale indicates expression level

We wondered what genes might be responsible for endothelial cell senescence. We screened endothelial cell senescence genes related to UM metastasis and prognosis using four gene sets, firstly, we screened endothelial cell-specific DEG (differential expressed genes) using log2fd higher than 0.5 as the threshold, and then we downloaded senescence-promoting gene sets from the cellage website [23], and also screened prognostic-related genes of UM using COX regression analysis, and according to statistical significance the first 5000 were taken, last, the endothelial cell cluster specific genes were intersected with the first three gene sets to obtain 3 genes (Fig. 3 A), which were displayed on the volcano plot of endothelial cell differential genes (Fig. 3B). We displayed the expression of these 3 genes in each cell types. We find the KLF4 expression was significantly higher in the endothelial cells and associated with prognosis (Fig. 3 C, 3D). we looked at the expression of KLF4 between primary and metastasis, KLF4 in the endothelial cells of metastatic tumors is significantly higher compared to primary, which implies that KLF4 may be a specific senescence driver in the endothelial cells (Fig. 3F and G). KLF4 is a very important transcription factor with a major role in cell cycle regulation and cell differentiation [24]. Particularly, we found that as early as 2006, there was literature reporting that KLF4 drives P21 to block the cell cycle without affecting P16 levels, consistent with our analysis results (Fig. 2 C). We arranged expression heatmaps for 80 TCGA samples of KLF4 and two other genes according to their metastatic status, revealing a more consistent correlation of high KLF4 expression with metastasis (Figure. S1A). We further incorporated the expression levels of these three genes into an artificial neural network for training and compared their accuracy in predicting metastasis, finding KLF4 significantly outperforming the others (Figure. S1B, S1C) [25].

Overall, KLF4 is a senescence-driving transcription factor specifically expressed in endothelial cells and was significantly associated with prognosis and metastasis as seen on 80 samples at the bulk level of TCGA. However, we still lack evidence that KLF4-driven senescent cells directly promote tumor migration.

### Up-regulation of KLF4 in the senescent endothelial cells of metastatic UM patients

**Fig. 4:**
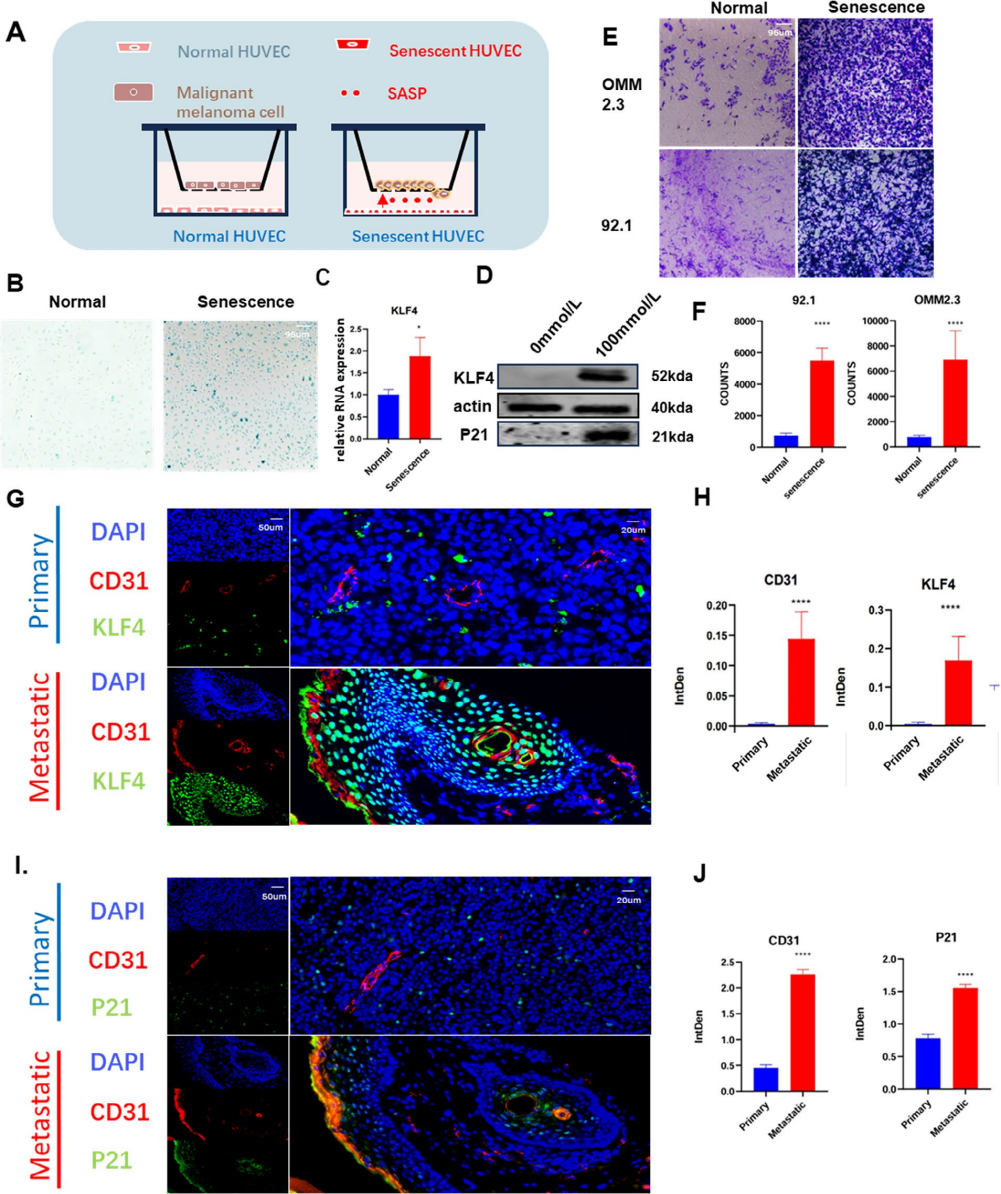
The expression of KLF4 is elevated in senescent endothelial cells of metastatic patients. (**A**) A model of tumor senescence endothelial stimulation of tumor cell migration. The senescent cells are induced by H2O2(100mmol/L) (**B**) Identification of senescent cells by SAβ-gal staining between H2O2-induced senescent HUVECs and normal HUVECs. (**C**) Relative RNA expression of KLF4 and P21 between H2O2-induced(100mmol/L) senescent HUVECs and normal. (**D**) Western blot showing the expression of KLF4 and P21 between H2O2-induced(100mmol/L) senescent HUVECs and normal. (**E**) Transwell experiments showing the effects of senescent HUVECs on cell migration and invasion in 92.1 and OMM2.3. (**F**) Statistical analysis of the number of cells migrating in 92.1 and omm2.3. (**G**) Images of multiplex in situ hybridization with immunofluorescence showing the KLF4 and CD31 in primary and metastasis ocular melanoma. (**H**) Statistical analysis of the intden of KLF4 and CD31 between metastatic and primary cells. (**I**) Images of multiplex in situ hybridization with immunofluorescence showing the P21 and CD31 in primary and metastasis ocular melanoma. (**J**) Statistical analysis of the intden of KLF4 and CD31 between metastatic and primary cells

To verified the relationship between KLF4 expression and endothelial cell senescence, and how them affect tumor cells, we firstly build an in vitro model (Fig. 4A). We induced endothelial cell senescence using hydrogen peroxide and combined P21 (Fig. 2D) with SA- β- Gal staining (Fig. 4B) confirms the senescence status of cells [26]. H2O2-induced senescent HUVECs exhibit significant KLF4 and P21 transcriptional and translational activation (Fig. 4 C, 4D). Using Transwell to simulate the in vivo microenvironment of tumors, 92.1 and OMM2.3 uveal melanoma cell lines were co cultured with senescent endothelial cells in a Transwell chamber [27]. After 24 h, the migration status of the cells was observed, and it was found that the migration of tumor cells co cultured with senescent endothelial cells significantly increased (Fig. 4E, 4 F). The above validation illustrates the presence of significant upregulation of KLF4 in senescent endothelium and that this upregulation is strongly associated with metastasis. To further confirm this, we took tissue sections of human uveal melanoma for in situ immunofluorescence staining. Significantly increased levels of KLF4 and CD31 in metastatic patients were clearly seen on the sections (Fig. 4G and H). The function of KLF4 to induce P21 expression to arrest the cell cycle has been discovered previously by scientists [6]. We further stain the P21 and CD31 to confirm whether liver endothelial cells undergo periods of senescence during remodeling. We find the endothelial cells in patients with metastasis had obvious senescent (Fig. 4I, 4 J).

Combining the above results we could find that the endothelial cells of metastatic tumors in UM patients showed significant senescence changes, and KLF4 was significantly elevated in the senescent endothelium of UM patients, and this KLF4 overexpression in senescent endothelium was associated with the promotion of tumor metastasis.

### KLF4-induced senescent endothelium promoted tumor cell metastasis by CXCL12

**Fig. 5:**
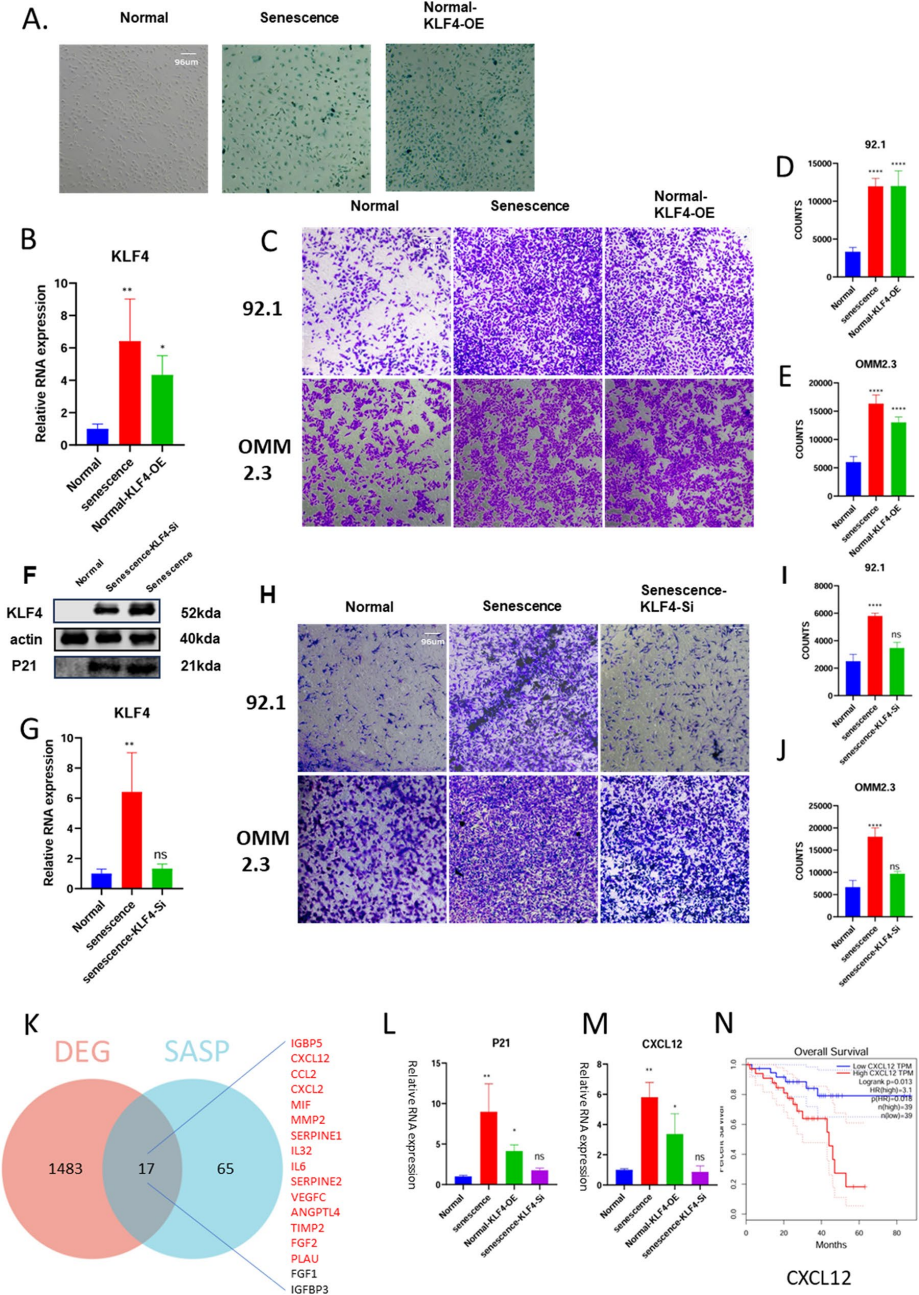
KLF4- induced senescent HUVECs secrete CXCL12 to prompt the migration of tumor cells. (**A**) Identification of senescent cells by SAβ-gal staining between H2O2-induced senescent HUVECs normal HUVECs and normal KLF4-OE. (**B**) Relative RNA expression of KLF4 and P21 between senescent HUVECs, normal KLF4-OE and normal HUVECs. (**C**) Transwell experiments showing the effects of normal KLF4-OE and senescent HUVECs on cell migration and invasion in 92.1 and OMM2.3. (**D-E**) Statistical analysis of the number of cells migrating in 92.1 and omm2.3. (**F**) Western blotshowing the expression of KLF4 and P21 between senescent HUVECs, Senescence KLF4-Si and normal HUVECs. (**G**) Relative RNA expression of KLF4 and P21 between senescent HUVECs, Senescence KLF4-Si and normal HUVECs. (**H**) Transwell experiments showing the effects of Senescence KLF4-Si senescent HUVECs on cell migration and invasion in 92.1 and OMM2.3. (**I-J**) Statistical analysis of the number of cells migrating in 92.1 and omm2.3. (**K**) Venn diagram the overlap between DEG (differential genes in metastatic tumor endothelium versus in situ endothelium in single-cell sequencing, *p* < .05) and SASP (red: up-regulated; black: down-regulated). (**L-M**) Relative RNA expression of P21 and CXCL12 between normal HUVECs, senescent HUVECs, normal KLF4-OE and Senescence KLF4-Si. (**N**) Prognosis of CXCL12 in UM. (*p* < .0001, ****; *p* < .01, **; *p* < .05, *; ns, no significance, t-test)

To further demonstrate whether it was H2O2-induced overexpression of KLF4 that caused endothelial cell senescence, we overexpressed KLF4 on normal HUVECs, which demonstrated that HUVECs overexpressing KLF4 showed similar phenotype (Fig. 5A, B) and tumor-promoting migration ability (Fig. 5C, D, E) to H2O2-induced senescent HUVECs, and then we further knocked down KLF4 in senescent HUVECs, and the senescent HUVECs lost their senescent phenotypes (Fig. 5F, G) and tumor-promoting migration ability (Fig. 5H, I, J). Senescent endothelium generally has the ability to secrete SASP, and the ability of senescent endothelium to secrete SASP to promote tumor cell migration has been verified in different tumors [8], we make sure that the ability of HUVECs to promote tumor migration is derived from their SASP secretion, and so we identified the SASP genes that are significantly overexpressed among the endothelial cell differential genes obtained by single-cell sequencing: CXCL12, CCL2 and IGFBP5 (Fig. 5K, S2A). We targeted C-X-C motif chemokine ligand 12 (CXCL12), the SASP with the highest expression in senescent endothelial cells that also has the greatest impact on UM, by combining real-time fluorescence quantitative PCR measurements of SASP expression in senescent HUVECs (Figure. S2B, S2C). And further determined that the expression of CXCL12 in HUVECs was consistent with the expression of KLF4 and p21 (Fig. 5B, G, L, M). Combined with the evidence from previous studies [28] and the clinical prognosis of different SASP in UM (Fig. 5N, S2D), we confirmed that CXCL12 is a key factor leading to the promotion of tumor cell migration in senescent endothelial cells.

In conclusion, we demonstrate that KLF4 is a key gene driving senescence in UM endothelial cells, and overexpression of KLF4 in normal endothelial cells induces senescence and promotes tumor cell migration, whereas knockdown of KLF4 in such senescent endothelial cells inhibits their senescence, and the ability to promote tumor cell migration disappears. Combining single-cell sequencing and PCR, we defined the SASP composition of senescent endothelial cells and identified the chemokine CXCL12 as the most critical secreted protein among them.

### Senescent endothelial cells regulate the tumor microenvironment for UM metastasis

**Fig. 6:**
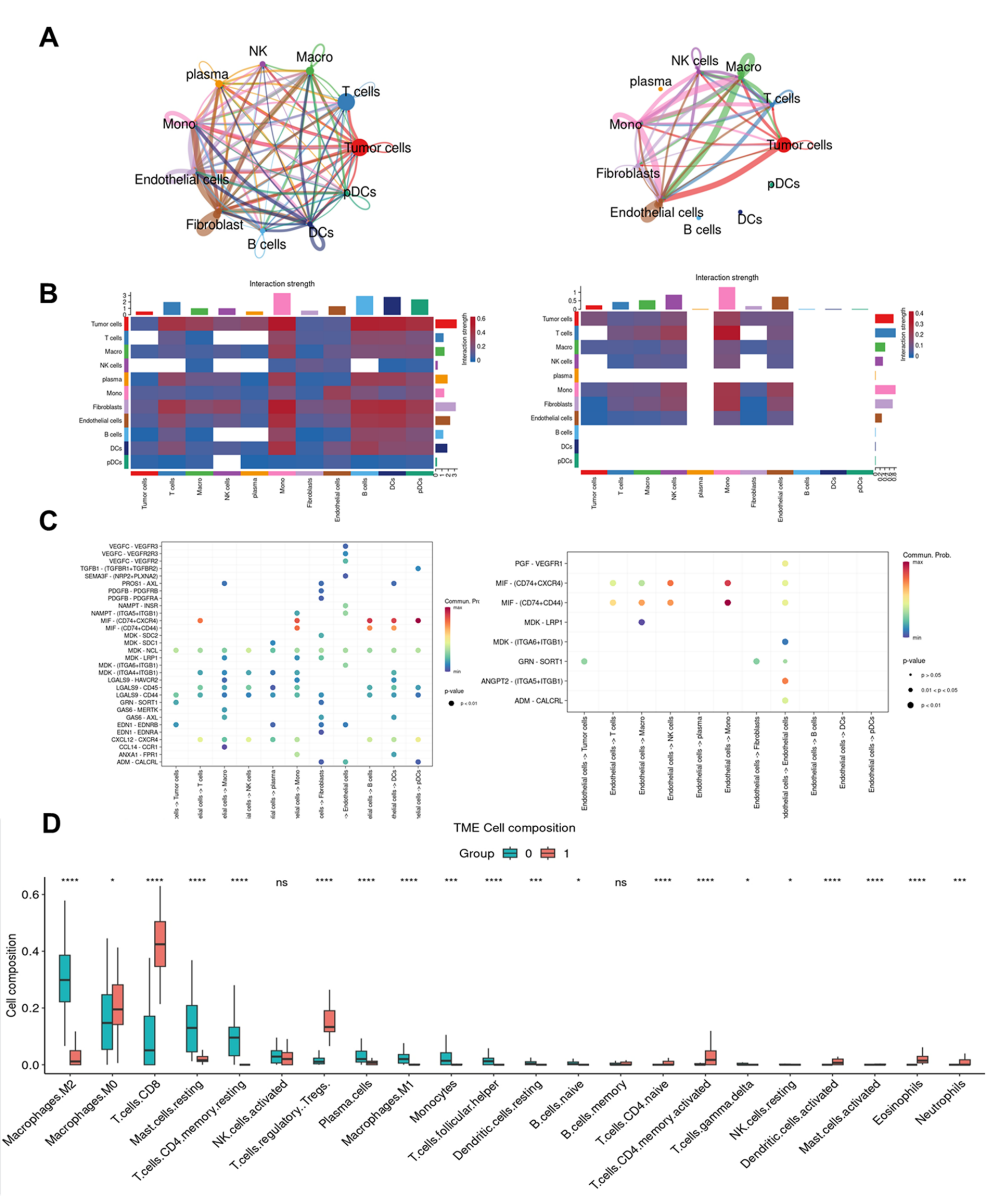
Crosstalk between endothelial cells with the other clusters. (**A**) Circle plots (by CellChat analysis) depict the differential interaction strength in the cell-cell communication network between metastatic (left) and primary (right). Color type represents cell type, and line thickness represents interaction strength (the thicker, the stronger). (**B**) Heatmap (by CellChat analysis) depict the differential interaction strength in the cell-cell communication network between metastatic (left) and primary (right). Color scale indicates interaction strength. (**C**) Dot plots (analyzed by CellChat) depict the signaling pathways of endothelial cells acting on other cell types in the cell-cell communication network between metastatic (left) and primary (right). Color scale indicates expression level. (**D**) Boxplot: boxplot of estimated proportions of different cell types of bulk RNA calculated in CIBERSORTx based on LM22, and show differences in different subtypes of immune cells in UM. The color indicates the group (*p* < .0001, ****; *p* < .01, ***; *p* < .05, *; ns, no significance, t-test)

We tried to figure out what role endothelial cells play in the tumor microenvironment. We used the cellchat package to group the samples and analyzed the crosstalk between endothelial cells and other cells in the tumor by comparing in situ and metastatic cancers [29] (Fig. 6A and B), and found that the endothelial cells’ ligand-receptor interactions with other cells in metastatic cancers were significantly higher than those in in situ cancers in both number and strength (Fig. 6A and B). We further compared the ligand-receptor interactions between endothelial cells and other cells and found that the MIF pathway had a significant effect on immune cells within metastatic tumors, especially monocytes and T cells [30] (Fig. 6 C). We combined previous research to analyze the T cell composition in UM and found that there are many Treg cells in metastasis UM (Fig. S3C, S3D). Furthermore, our results based on TCGA data deconvolution also showed that endothelial cells have a significant correlation with Treg cells and exhausted CD8 + T cells [31] (Fig. S3A), and the Treg cells in metastatic tumors are significantly more than those in situ tumors (Fig. 6D). This means that endothelial cells in metastatic tumors participate in the formation of tumor immune suppression microenvironment. The involvement of endothelial cells in the process of immune suppression is predominantly mediated by MIF, which is a type of SASP that often increase in inflammation-induced senescence [32]. We posit that the potent effect of endothelial cells on immune cells is attributable to the role of senescent endothelial cells.

In summary, these results suggested the crucial role of endothelial cells in promoting an immune-suppressive microenvironment within metastatic tumors. By analyzing cellular crosstalk and ligand-receptor interactions with CellChat and CIBERSORTx, we discovered that these cells significantly enhance communication with immune cells, particularly Treg cells. These findings underscore the potential of targeting endothelial cells in strategies aimed at improving tumor microenvironment.

## Discussion

In conclusion, this study highlighted the role of endothelial cell in UM liver metastasis. Firstly, the populations of endothelial cells in liver metastasis are much more than primary UM. Moreover, metastatic UM endothelial cells exhibited a abnormal senescent status, which was due to the upregulation of key senescent factor KLF4. Senescent endothelial cells consequently release SASP factors, which contribute to tumor cell recruitment and metastasis to liver, and in the SASP secreted by senescent endothelial cells, the chemokine CXCL12 is critical for senescent endothelium leading to tumor cell migration (Graphic Abstract).

Accumulating evidence supports the role of SASP in enhancing tumor cell migration within the tumor microenvironment [8, 33, 34]. Our study is based on previously published single-cell data on UM [6, 9, 12], which provide comprehensive insights into the metastatic mechanism of uveal melanoma, focusing on driver genes, immunosuppression, and tumor heterogeneity. We collected 10X single-cell sequencing data from all UM metastatic patients that are currently publicly available at GEO, i.e., the five existing metastatic patients, and compared them with single-cell sequencing data from six in situ patients. This study is the first to analyze the endothelial information of uveal melanoma based on single-cell data and found that the endothelial cells in uveal melanoma metastasis has a significant damage status, which is a good guideline for the treatment of metastatic patients, and the removal of senescent endothelial cells in hepatic metastasis may be an important way to inhibit the development of metastasis.

Tumor stromal cells consist of fibroblasts and endothelial cells. While the functions of endothelial cells have been extensively studied, we have limited knowledge about the role of senescent endothelial cells in the tumor microenvironment and their direct impact on cancer metastasis. Our study addresses this knowledge gap by analyzing single-cell RNA data from clinical patients, demonstrating that senescent endothelial cells are prevalent in metastatic uveal melanoma tumors and can promote tumor cell metastasis. We have also identified KLF4 as a potential regulator of this process. Our laboratory experiments have further supported these conclusions by simulating the process of senescent endothelial cells driving tumor metastasis. Given the relationship between cellular senescence and epigenetics [35], the regulation of endothelial cell senescence by KLF4 may be related to epigenetics, it may be necessary for future research to delve deeper into the specific mechanisms.

Our most exciting finding is the discovery in single-cell sequencing results that metastatic tumor endothelial cells have a significant senescent state that is driven by KLF4 and validated in human tissues with in vitro experiments, and confirmed by in vitro modeling based on existing studies [8]. KLF4 is a key transcription factor known for inducing cellular senescence. Our study builds upon the early findings of KLF4, highlighting its role in upregulating P21 to arrest the cell cycle and inhibit cell growth without affecting the expression of P16 [19]. KLF4 is upregulated in response to mild DNA damage, leading to cell cycle arrest and the subsequent formation of senescent cells. However, excessive DNA damage can result in apoptosis. We also find different cell types who exhibit high expression of KLF4 usually accompanied by an increase in P21 in various tumors. This suggests that KLF4 may induce senescence in a broader range of tissues.

The key to senescent cell-induced tumor metastasis is SASP secreted by senescent cells, and we confirmed that the most critical component of the SASP leading to metastasis in uveal melanoma is CXCL12, a chemokine that has been widely shown to promote tumor metastasis. Senescent endothelial cells significantly promote tumor cell migration by secreting CXCL12, which has been demonstrated by a large body of previous evidence [28] combined with our single-cell sequencing validation results, but the receptor for CXCL12 targeting to uveal melanoma cells is still unclear, and the lack of a suitable in vivo model of UM for validation is an issue that needs to be addressed.

Interestingly, cellular senescence not only alters cellular phenotype but also triggers the secretion of SASP, which induces immune cell infiltration and promotes inflammation in tumor tissues. The infiltration of immune cells is a crucial factor influencing tumor prognosis. While uveal melanoma has been traditionally considered as a cold immune tumor with limited immune cell infiltration, our study suggests that uveal melanoma metastasis exhibit significant immune cell infiltration [6]. Analysis using Cellchat also revealed strong ligand-receptor interactions between endothelial cells and immune cells. The current focus of immunotherapy for uveal melanoma (UM) still revolves around understanding the mechanisms of immune suppression in UM. Considering that single-agent LAG3 immunotherapy has not yielded ideal results in melanoma, it is more suitable as an adjunct therapy to PD1 inhibition [36]. Therefore, the research on immunosuppressive agents for UM is expected to remain quite challenging.

Since senescent endothelium is so important in liver metastasis, how exactly can senescent cells be removed? There are already a lot of senescence removal drugs available, such as quercetin, anthocyanins, etc. In addition, some green nanomaterials targeting the liver may also be a good choice for removing senescent cells [37], Stem cell exosomes are inherently good at reversing [38], and exosomes, a class of liposomes, have very little adverse effect on the body at the same time [39], however, compared to traditional drugs, nanomaterials have been less experimented with in the clinic, and we may look forward to the prospect of green nanomaterials in cancer treatment [40].

In a word, Not only are there more endothelial cells in metastatic tumors, but also many senescent endothelial cells are present, and these senescent endothelial cells have a significant pro-metastatic effect on tumors. Our study still has certain limitations. We were unable to obtain endothelial data from the primary foci of metastatic patients and could only compare liver metastasis with endothelium from other in situ cancer patients, we hope that we can collect a sufficient number of paired samples from the same patient source to elucidate the genetic characteristics of tumor cells and non-tumor cells, conduct detailed studies on the tumor microenvironment in metastatic uveal melanoma (UM), and clarify the functions of stromal cells and immune cells.

### Electronic supplementary material

Below is the link to the electronic supplementary material.

**Additional file 1. Figure S1**: Predict the prognosis of PM by KLF4 in artificial neural network model.

**Additional file 2. Figure S2**: Defining SASP in senescent endothelial cells.

**Additional file 3. Figure S3**: Endothelial cells promote tumor immunosuppressive microenvironment formation.

## Data Availability

The datasets used and analysed during the current study are available from the corresponding author on reasonable request.

## Abbreviations

CCL2: The C-C motif chemokine ligand 2
CXCL12: C-X-C motif chemokine ligand 12
DEG: Differentially Expressed Gene
DC: Dendritic Cell
DDRTree: Discriminative Dimensionality Reduction Tree
EC: Endothelial Cell
FDR: False Discovery Rate
FC: Fold Change
GEO: Gene Expression Omnibus
GSVA: Gene Set Variation Analysis
IGBP5: Insulin Like Growth Factor Binding Protein 5
IGHG1: Immunoglobulin Heavy Constant Gamma 1
KLF4: Kruppel-Like Factor 4
KEGG: Kyoto Encyclopedia of Genes and Genomes
LAG3: Lymphocyte Activation Gene 3
LM22: Leukocyte Matrix 22
MLANA: Melanoma Antigen Recognized by T Cells 1
MIF: Macrophage Migration Inhibitory Factor
MITF: Microphthalmia-associated Transcription Factor
NK: Natural Killer
PCA: Principal Component Analysis
PD1: Programmed cell death protein 1
pDC: Plasmacytoid Dendritic Cell
PPI: Protein-Protein Interaction
SASP: Senescence-Associated Secretory Phenotype
SD: Standard Deviation
SEM: Standard Error of Mean
TCGA: The Cancer Genome Atlas
TGF-β: Transforming Growth Factor Beta
UMAP: Uniform Manifold Approximation and Projection
UM: Uveal Melanoma
VWF: Von Willebrand Factor
