# Supplementary material for: Senescent endothelial cells promote liver metastasis of uveal melanoma in single-cell resolution
Source: J Transl Med. 2024 Jul 1;22:605. doi: 10.1186/s12967-024-05430-1 (PMC11218175; doi:10.1186/s12967-024-05430-1)
Supplement: Supplementary file 1 — Additional file 1. Figure S1: Predict the prognosis of PM by KLF4 in artificial neural network model. [file 12967_2024_5430_MOESM1_ESM.docx]

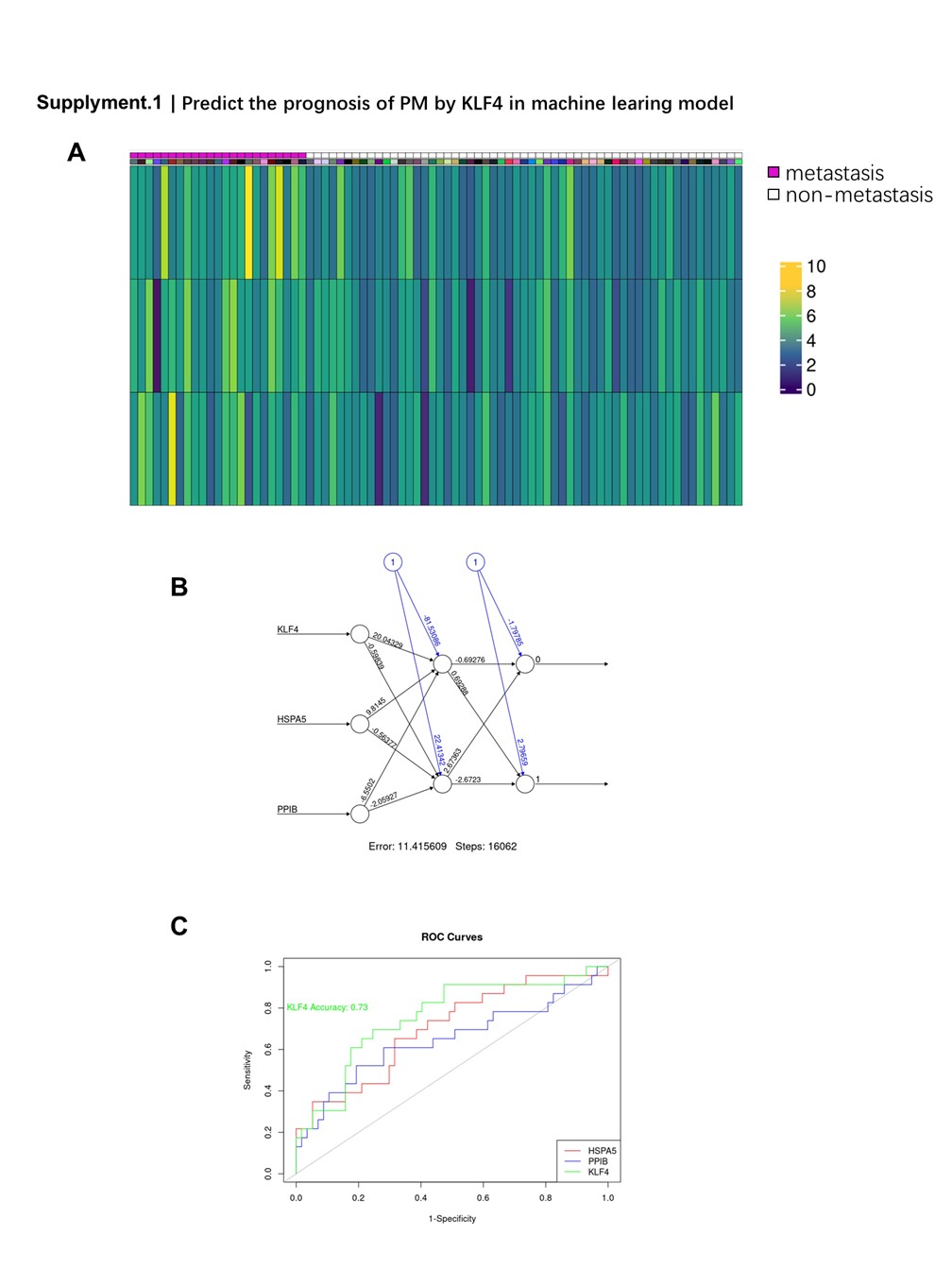


**Figure S1: Predict the prognosis of PM by KLF4 in artificial neural netwwork model;**

(A) Heatmap: expression of overlap genes in metastatic and non-metastatic UM. (B) Results of artificial neural network visualization. (C) ROC curve of artificial neural network.

senescent HUVECs, KLF4-SI and normal HUVECs. (D) Prognosis of CCL2 in UM. (p < .0001, ****; p < .01, **; p < .05, *; ns, no significance, t‐test)
