# Supplementary material for: Senescent endothelial cells promote liver metastasis of uveal melanoma in single-cell resolution
Source: J Transl Med. 2024 Jul 1;22:605. doi: 10.1186/s12967-024-05430-1 (PMC11218175; doi:10.1186/s12967-024-05430-1)
Supplement: Supplementary file 2 — Additional file 2. Figure S2: Defining SASP in senescent endothelial cells. [file 12967_2024_5430_MOESM2_ESM.docx]

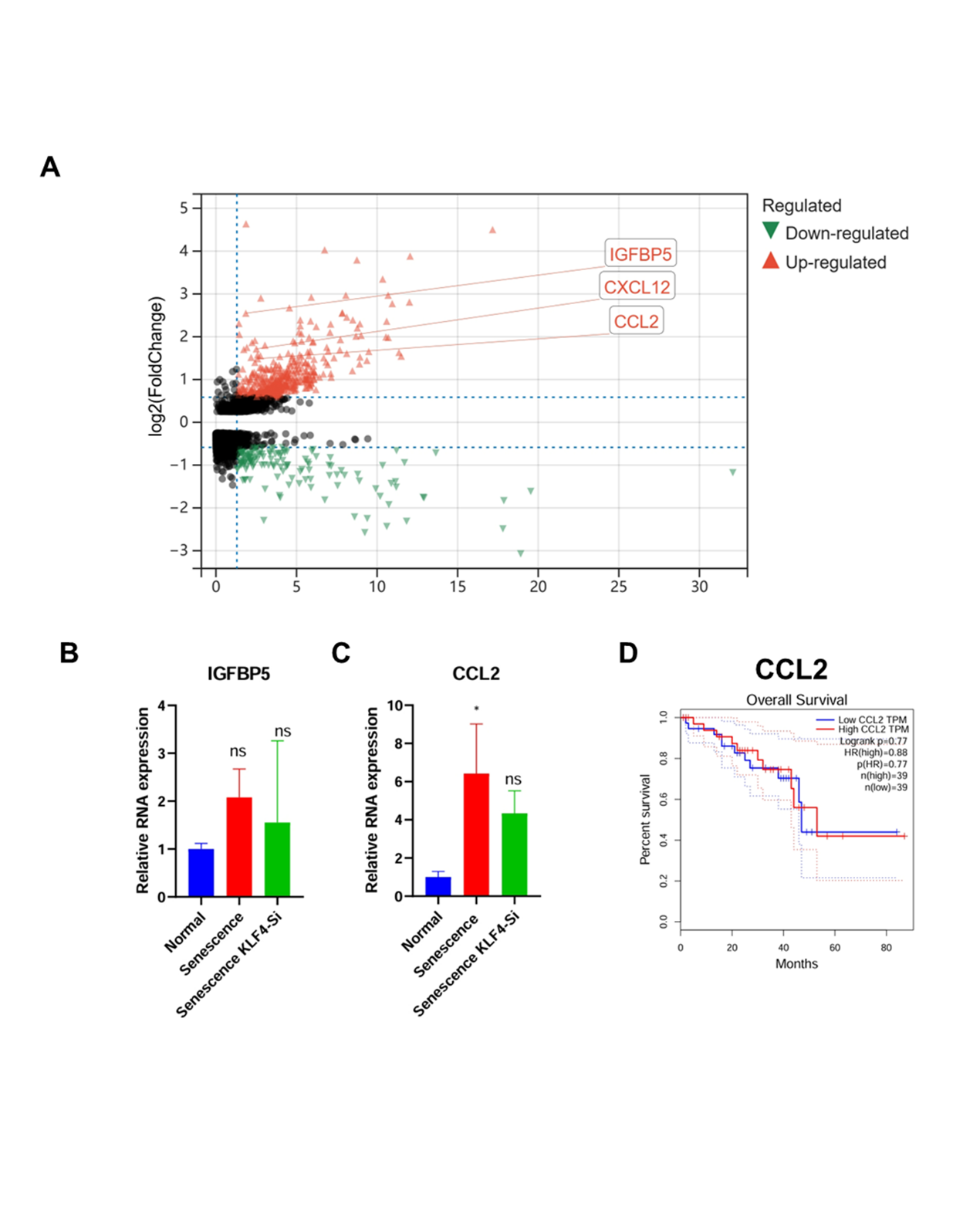


**Figure S2: Defining SASP in senescent endothelial cells**

(A) Volcano map showing expression of genes in overlap (CXCL12, IGFBP5, CCL2) in endothelial cells of DEG. (B) Relative RNA expression of IGFBP5 between senescent HUVECs, KLF4-SI and normal HUVECs. (C) Relative RNA expression of CCL2 between senescent HUVECs, KLF4-SI and normal HUVECs. (D) Prognosis of CCL2 in UM. (p < .0001, ****; p < .01, **; p < .05, *; ns, no significance, t‐test).
