# Supplementary material for: Senescent endothelial cells promote liver metastasis of uveal melanoma in single-cell resolution
Source: J Transl Med. 2024 Jul 1;22:605. doi: 10.1186/s12967-024-05430-1 (PMC11218175; doi:10.1186/s12967-024-05430-1)
Supplement: Supplementary file 3 — Additional file 3. Figure S3: Endothelial cells promote tumor immunosuppressive microenvironment formation. [file 12967_2024_5430_MOESM3_ESM.docx]

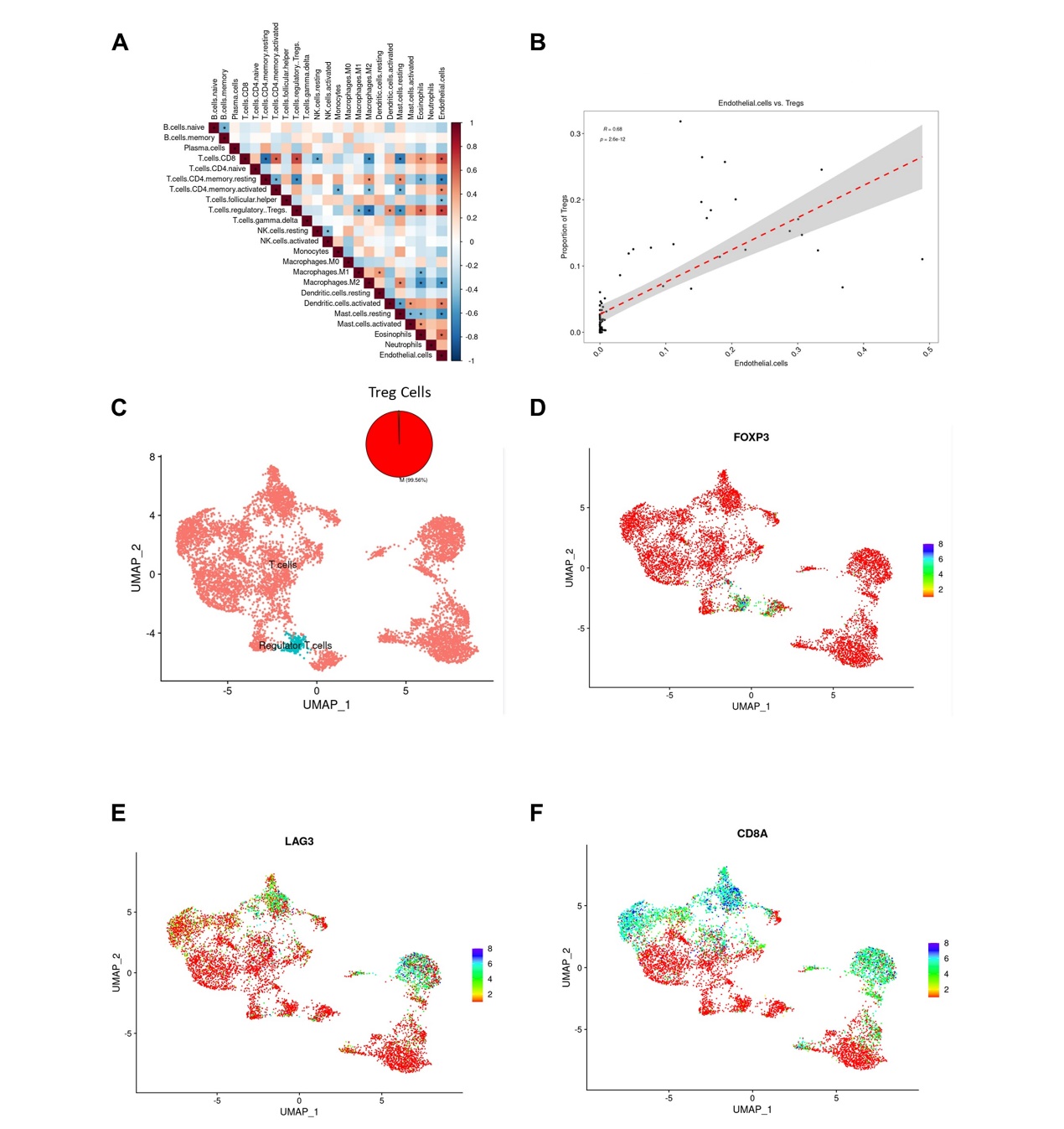
 **Figure S3: Endothelial cells promote tumor immunosuppressive microenvironment formation;**

(A) Signature score heatmap: signature score of bulk RNA data calculated in CIBERSORTx based on LM22. (p < .05, *; person). (B) Correlation analysis depicting the relationship between Treg cells and endothelial cells, which calculated based on CIBERSORTX (p = 2.6e-12, *; spearman). (C) UMAP visualization of Treg cells. (D) Feature plots of total scores of marker genes of Treg cells. The color indicates the module scores of marker genes of different lineages. The pie chart shows the proportion of Treg cells in metastatic and non-metastatic uveal melanoma. (M= metastasis)
